# Perceptions and Barriers to Human Papillomavirus Vaccination and Cervical Cancer Screenings: A Survey Study of Underserved Populations in North Texas

**DOI:** 10.1002/puh2.70018

**Published:** 2024-12-18

**Authors:** Sofia Eva Olsson, Sameep Shah, Erin Haase, Kelly Pagidas

**Affiliations:** ^1^ Anne Burnett Marion School of Medicine Texas Christian University Fort Worth Texas USA; ^2^ Department of Computer Science College of Science and Engineering Texas Christian University Fort Worth Texas USA; ^3^ School of Medicine Louisiana State University Health Sciences Center Shreveport Louisiana USA

**Keywords:** barriers to care, barriers to healthcare, cervical cancer screening, human papillomavirus (HPV) vaccination, human papillomavirus, Pap smear, women's health

## Abstract

**Background:**

Human papillomavirus (HPV) is a common cause of cervical cancer along with several other neoplasms. With the availability of HPV vaccination and cervical cancer screenings, it is more likely for cervical cancers to be prevented or caught early in their course. However, there are disparities and barriers preventing all individuals from obtaining proper preventative care.

**Methods:**

An online survey analyzing compliance, barriers, and subjective perceptions of HPV vaccination and cervical cancer screening was distributed via flyers. These were placed in the half of zip codes with lowest median income in Fort Worth, Texas.

**Results:**

Respondents with non‐White race, income of $40,000 or less, and a maximum education of high school or less were 4.24, 3.2, and 1.2 times more likely to have incomplete HPV vaccination, respectively. Respondents with an income of $40,000 or less and a maximum education of high school were 3.2 and 2.6 times more likely to be overdue for cervical cancer screening, respectively. The most common barrier for HPV vaccination was lack of knowledge, and for cervical cancer screening, it was cost. Most respondents felt as though cervical cancer screening allows for early cancer detection and decreased mortality but also endorsed feelings of pain, embarrassment, and fear of the test.

**Conclusions:**

HPV vaccine and cervical cancer screening non‐compliance is likely multifactorial, including lack of knowledge and cost. Interventions such as increased access to pediatric care and increased opportunities for free preventative healthcare may be helpful in both reducing stigma and improving access to care.

## Graphical abstract

The most common barrier for HPV vaccination in North Texas was lack of knowledge, and for cervical cancer screening, it was cost. Community members of non‐White race, income of $40,000 or lower, and a maximum education of high school or lower were more likely to have incomplete HPV vaccination and overdue cervical cancer screening.

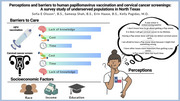

## 1 Introduction

Human papillomavirus (HPV) is the most common sexually transmitted infection with an estimated 85% of adults being exposed in their lifetime [1, 2]. Oncogenic strains of HPV are the cause of several anogenital and oropharyngeal carcinomas with cervical carcinoma being the most prevalent HPV‐associated cancer [3]. Additionally, the virus is associated with benign conditions, such as anogenital warts and laryngeal papillomatosis [3]. The HPV vaccine has demonstrated nearly 100% efficacy in preventing cervical oncogenesis if administered prior to HPV exposure [1]. In 2022, the World Health Organization (WHO) identified prophylactic HPV vaccination as a foundational pillar of the WHO cervical cancer elimination strategy [3]. They estimated that its implementation alongside cervical cancer screening could prevent 60 million cases of cervical cancer and 45 million deaths in the next century [3, 4, 5]. In the context of combating cervical cancer, both HPV vaccination and cervical cancer screenings serve as pivotal preventive measures [6].

In Texas, the HPV vaccination rates among female individuals aged 13–17 are approximately 54.8%, whereas the national average stands at 61.7% [7]. Ultimately, both in Texas and nationally, vaccinations are below the US goal of at least 80% of individuals obtaining complete immunization [8]. This variance suggests opportunities for refinement within preventive healthcare initiatives. Furthermore, the importance of early cervical cancer detection through screenings cannot be overstated. Regular Papanicolaou smear (Pap) tests, recommended every 3 years for women aged 21–65, play a pivotal role in identifying cervical cell dysplasia prior to the development of a carcinoma [9]. A systematic review of cervical cancer screenings in Europe determined that appropriate screening has led to a 41%–92% reduction in mortality among women [10].

With development and distribution of HPV vaccination and Pap smears, cervical cancer incidence and mortality rates have decreased by more than half since the mid‐1970's [11]. Major medical organizations, such as the American Cancer Society (ACS), US Preventative Services Task Force (USPSTF), and American College of Obstetricians and Gynecologists (ACOG), endorse both HPV vaccination and Pap smears as methods for reducing cervical cancer in the United States [12, 13, 14, 15, 16].

With a population mirroring national averages in key metrics such as gender distribution, educational attainment, and poverty levels, Fort Worth, Texas, presents a microcosm of the nation's demographic landscape [17, 18]. Therefore, the examination of the barriers to cervical cancer screenings and HPV vaccination in Fort Worth, Texas, presents valuable insights that may reflect patient needs at the state or national level. By dissecting the impediments preventing patients from accessing crucial healthcare services locally, strategies for addressing similar challenges nationwide can be considered.

To bridge these gaps and address healthcare disparities effectively, a targeted approach is imperative. By pinpointing specific barriers to screening services, tailored interventions can be crafted to meet the unique needs of Fort Worth's population. Moreover, leveraging the demographic parallels between North Texas and broader geographic regions, insights derived from local data offer guidance in formulating strategies aimed at promoting nationwide utilization of preventive care services.

## 2 Methods

### 2.1 Data Collection

A survey designed using Qualtrics software was used to gather data on individual barriers and perceptions regarding HPV vaccination and cervical cancer screenings in underserved Fort Worth areas. The survey was available in both English and Spanish. A team of research volunteers distributed flyers containing a quick response (QR) code to the survey in public locations within the half of Fort Worth's zip codes with lowest median household income, as identified by the 2017–2021 census [19]. Volunteers were instructed to prioritize flyer distribution in essential, low‐cost establishments such as laundromats, local grocery stores, community centers, and food pantries, while avoiding non‐essential or luxury venues like restaurants, coffee shops, clothing stores, and high‐end grocery outlets. After a 2‐week flyer distribution period, data collection was conducted over a 1‐year span, from November 2022 to November 2023.

Participants were eligible for the study if they indicated that their sex assigned at birth was female and were 18 years or older at the time of the survey. Informed consent was confirmed via a statement at the beginning of the survey.

### 2.2 Statistical Analysis

Compliance was determined on the basis of the Centers for Disease Control and Prevention (CDC) guidelines stating that HPV vaccination is recommended for individuals aged 9–14 years, administered as a two‐dose series [8]. Compliance for cervical cancer screenings was defined as having a screening within the past 3 years, based on guidelines from the ACOG [20].

Descriptive statistics were conducted on variables, including patient age, household income, education level, ethnicity, language preference, vaccination compliance, screening compliance, and perceptions. Odds ratios were calculated to identify disparities in HPV vaccination and cervical cancer screening across various demographic groups.

## 3 Results

A total of 72 individuals completed the survey and were included in this study. A total of 65 (90.28%) of these respondents completed the survey in English, whereas 7 (9.72%) completed the survey in Spanish (Table 1). The mean age of respondents was 34.98 years (standard deviation [SD] = 11.99), and mean household income was $41,548.61 (SD = 46,890.50). High school was most frequently selected as the highest level of education (*n* = 28; 38.9%), and most respondents identified with Hispanic or Latino race (*n* = 38; 52.8%).

**TABLE 1:** Demographic distribution of survey respondents.

Age	18–21 years old	21–39 years old	40–44 years old	45–54 years old	55–65 years old	Over 65 years old		
Number of individuals	13	31	18	2	8	0		
**Household income**	**$10,000 or less**	**$11,000–$40,000**	**$41,000–$50,000**	**$51,000–$70,000**	**$71,000–$90,000**	**$91,000–$110,000**	**$110,000–$199,000**	**$200,000 or more**
Number of individuals	22	25	5	8	4	3	2	3
**Highest level of education**	**Did not complete elementary school**	**High school**	**Some university/college**	**Graduated university**	**Masters degree**	**Doctoral degree**		
Number of individuals	3	28	18	16	5	2		
**Ethnicity**	**Black or African American**	**Hispanic or Latino**	**Native American or Alaskan**	**White or Caucasian**	**“Other”**			
Number of individuals	13	38	1	14	5			

### 3.1 HPV Vaccination

Of all respondents, 57.8% (*n* = 37) reported to have never been vaccinated for HPV, 14% (*n* = 9) received one dose, and 28.1% (*n* = 18) reported complete HPV vaccination with two or more doses administered.

Non‐White races were 4.24 times more likely to have incomplete HPV vaccination status (95% [CI 1.18, 15.23]) compared to their White or Caucasian counterparts. Respondents with an income level of $40,000 or lower were 3.2 times more likely to have incomplete HPV vaccination (95% CI [1.03, 9.82]). Those with a highest level of education being high school or lower were 1.2 times more likely to be incompletely vaccinated against HPV (95% CI [0.40, 3.68]).

The barriers to HPV vaccination were reported as lack of knowledge (*n* = 19; 39.58%), cost (*n* = 11; 22.91%), lack of time (*n* = 5; 10.41%), language barrier (2.08%, *n* = 1), transportation (*n* = 12.08%), and “other” (22.91%, *n* = 11).

### 3.2 Cervical Cancer Screening

There was a total of 46 respondents eligible for cervical cancer screening at 21–65 years of age. Of these individuals, 13.0% (*n* = 6) had never had a cervical cancer screening, 26.1% (*n* = 12) had a cervical cancer screening performed previously but were overdue at the time of the survey, and 60.9% (*n* = 28) were up to date on their cervical cancer screening as per ACOG guidelines.

Respondents with an income of $40,000 or lower were 3.2 times more likely to have never had a cervical cancer screening (95% CI [0.34, 29.23]) and 2.6 times more likely to be behind on cervical cancer screening (95% CI [0.58, 11.69]) as per ACOG guidelines. Respondents with their highest level of education of being high school or lower were 1.9 times more likely to have never had a cervical cancer screening (95% CI [0.34, 10.36]) and 1.5 times more likely to be overdue for cervical cancer screening (95% [CI 0.34, 6.55]).

The barriers to cervical cancer screening were cost (*n* = 21; 45.65%), lack of time (*n* = 12; 26.08%), lack of knowledge (*n* = 5; 10.87%), transportation (*n* = 3; 6.52%), and “other” (*n* = 11; 23.91%). No respondents selected language barriers as an inhibitor to cervical cancer screening.

### 3.3 Perceptions

A total of 34.5% (*n* = 19) of respondents selected that it is very likely or somewhat likely that they will develop cervical cancer in their lifetime, and 25.5% (*n* = 14) believed that it is very likely or somewhat likely that they will develop cervical cancer in the next few years (Table 2). A majority (*n* = 40; 72.7%) of respondents agreed that cervical cancer screenings are very likely to help detect cervical cancer early, and 69.1% (*n* = 38) believe this test is very likely to decrease chances of dying from cervical cancer. However, 42.6% (*n* = 23) reported that they are afraid or somewhat afraid of cervical cancer screening because they fear it might reveal something wrong. Additionally, 14.8% (*n* = 8) do not know how to go about scheduling a cervical cancer screening. A collective 5.6% (*n* = 3) believe this screening takes too much time, and 46.3% (*n* = 25) believe it is embarrassing and painful. Approximately a quarter (*n* = 15; 27.8%) of respondents stated that they have other issues more important than getting a cervical cancer screening.

**TABLE 2:** Percentage of responses for cervical cancer screening perceptions.

	Not likely	Somewhat likely	Very likely
It is likely I will get cervical cancer in my lifetime	58.49	33.96	7.55
It is likely I will get cervical cancer in the next few years	77.36	18.87	3.77
Having a Pap smear done will help me detect cervical cancer early	5.66	11.32	83.02
A Pap smear will decrease my chances of dying from cervical cancer	13.21	20.75	66.04
I am afraid to have a Pap smear done because I might find something wrong	59.62	30.77	9.62
I do not know how to go about getting a Pap smear	61.54	23.08	15.38
A Pap smear takes too much time	78.85	17.31	3.85
A Pap smear is embarrassing/painful	46.15	42.31	11.54
I have other issues more important than getting a pap smear	70.59	27.45	1.96

## 4 Conclusions

With only 28.1% of study participants having completed HPV vaccination and 60.9% being up to date on recommended cervical cancer screening, systemic interventions must be considered to better protect underserved communities. Compliance rates appeared to be far lower for HPV vaccination than Pap smears with the most common barrier being lack of knowledge rather than cost. This may be related to vaccine hesitancy that has increased dramatically since the SARS‐CoV‐2 pandemic in 2020 and is prevalent in the Southern US region [21, 22, 23]. A 2024 study identified that 23.1% of survey respondents representing 12 countries reported increased hesitancy toward routine vaccinations following the SARS‐CoV‐2 pandemic [24]. Additionally, vaccine hesitancy, along with mistrust of medical establishments, is more prevalent in non‐White communities [22, 25–28]. With the recommended timing of HPV vaccination being from ages 9 through 14, primary interventions must often begin with the patient's pediatrician. This requires open discussions and trust between patient guardians and pediatricians with an individualized approach yielding greater vaccine acceptance [29]. With an average respondent age of approximately 35 years, alternative settings, such as pharmacies, adult physician's offices, and gynecologic offices, may continue to improve accessibility to prophylactic HPV vaccination in all demographics. The present study focused on HPV vaccination among female community members due to the investigation of barriers to cervical cancer prevention. Importantly, the male population is equally recommended for prophylactic HPV vaccination to prevent male anogenital carcinoma, oropharyngeal carcinoma, and viral transfer to others [3].

The main barrier to cervical cancer screening was cost despite the preventative screening being covered by nearly all health insurance plans. However, 10.2% of US citizens and 20.5% of Texas residents are uninsured, which results in an average out‐of‐pocket price of $143 for these patients [30, 31]. However, it is important to note that many US cities organize free testing sites or events to support uninsured individuals, though disseminating these resources to those in need is a challenge. Overall, most respondents agreed that cervical cancer screenings effectively detect cancer and reduce mortality; however, nearly half of them found the test embarrassing, painful and were afraid of detecting a health issue. These sentiments, along with low compliance rates due to cost, collectively demonstrate a need for decreased stigma and improved free access to cervical cancer screening services.

This study found that participants with non‐White race, income less than $40,000, and a highest education of high school or below were more likely to be overdue for HPV vaccination and the latter two populations were more likely overdue for cervical cancer screening. Additionally, 58% of respondents reported that it was unlikely that they would develop cervical cancer in their lifetime, whereas 41% provided a response of “somewhat likely” or “very likely.” This is an interesting sentiment that may necessitate future research further analyzing the public's understanding of cervical cancer risk factors. These demographic groups must be targeted with related health initiatives in an attempt to provide better education, lower costs, and improved access to resources.

The present study is the first to identify patient compliance, barriers, and perceptions of HPV vaccination and cervical cancer screenings. There are several strengths to the study, such as the year‐long survey response period and the demographic similarities between Fort Worth, Texas, and the United States. Additionally, providing Spanish translations of the survey and flyer allowed for a more complete patient response profile. The use of QR codes for survey distribution also limited survey completion only to those with access to a mobile phone and the internet. In 2022, the WHO provided single‐dose recommendations for prophylactic HPV vaccination, reporting that a single dose may provide non‐inferior immunogenic protection to the multidose regimen [3]. Although this study follows the current ACOG, ACS, and USPSTF recommendations to define immunization compliance, we must recognize that a single dose of the HPV vaccine is superior to no vaccination [3, 12–16]. A three‐dose vaccination series is recommended for unvaccinated individuals over the age of 15 years [13]. This study did not include data on the timeline of respondent vaccination or compliance of the three‐dose vaccine regimen. Additionally, the data does not capture the option of HPV and cervical cancer co‐testing every 5 years after the age of 30 years [13]. We encourage further data collection in multiple US cities to identify geographic trends in cervical cancer screenings and HPV vaccination.

Overall, non‐White, lower income, and less educated individuals are more likely to have incomplete HPV vaccination status and non‐compliant cervical cancer screening. This is primarily due to lack of knowledge regarding HPV immunization and cost of cervical cancer screenings. The majority of respondents understood the importance of cervical cancer screenings, so efforts to improve access and dissemination of related resources are vital in vulnerable communities.

## Author Contributions

**Sofia Eva Olsson**: conceptualization, data curation, investigation, methodology, project administration, resources, writing of original draft, review, and editing. **Sameep Shah**: data curation, formal analysis, methodology, review, and editing. **Erin Haase**: investigation, writing of original draft, review, and editing. **Kelly Pagidas**: conceptualization, supervision, methodology, review, and editing.

## Ethics Statement

This study was submitted to and approved by the Texas Christian University Institutional Review Board with the identification number 2022‐149.

## Consent

Participants gave informed consent for participation in the study.

## Conflicts of Interest

The authors declare no conflicts of interest.

## Data Availability

The data that support the findings of this study are available from the corresponding author upon reasonable request.
